# The effect of emergency department pharmacists on drug overuse and drug underuse in patients with an ADE-related hospitalisation: a controlled intervention study

**DOI:** 10.1186/s12913-022-08696-7

**Published:** 2022-11-17

**Authors:** R. N. Rahman, B. Nikolik, M. A. J. de Ridder, A. E. Hoek, M. J. A. Janssen, S. C. E. Schuit, F. Karapinar-Çarkit, P. M. L. A. van den Bemt

**Affiliations:** 1 Department of Clinical Pharmacy and Pharmacology, University Medical Center Groningen, Groningen, The Netherlands; 2 Department of Hospital Pharmacy, Erasmus MC, University Medical Center Rotterdam, Rotterdam, The Netherlands; 3 Department of Clinical Pharmacy, OLVG Hospital, Amsterdam, The Netherlands; 4 Department of Medical Informatics, Erasmus MC, University Medical Center Rotterdam, Rotterdam, The Netherlands; 5 Department of Emergency Medicine, Erasmus MC, University Medical Center Rotterdam, Rotterdam, The Netherlands; 6 Board of Directors, University Medical Center Groningen, Groningen, The Netherlands

**Keywords:** ADE-related side effects and adverse reactions, Hospital emergency service, Medical drug overuse, Quality of health care, Prospective studies, Drug utilization review

## Abstract

**Background:**

Drug overuse or drug underuse are the most common causes of adverse drug events and can lead to hospital admissions. Using clinical pharmacists in the emergency department may improve patient safety as they are specialised in recognising of adverse drug events and tackling drug overuse and drug underuse. This study tested the effect of an emergency department pharmacist on the number of medication changes for drug overuse and drug underuse taking place in patients with an adverse drug event-related hospitalisation following an emergency department visit.

**Methods:**

A multicenter prospective non-randomized controlled intervention study was conducted in a university hospital and a general teaching hospital. Trained emergency department pharmacists included patients in the intervention group with a hospital admission related to an adverse drug event. The interdisciplinary intervention consisted of a pharmacist-led medication review, patient counselling regarding medication, and information transmission to general practitioners and community pharmacies after discharge. The control patients were also admitted after an emergency department visit and received the usual care. The primary outcome was the number of medication changes for drug overuse and drug underuse that took place during hospital admission and persisted 6 months thereafter. Poisson regression analysis was used to estimate the difference in these medication changes between the intervention group and the control group.

**Results:**

A total of 216 patients were included (intervention group 104, control group 112). In the intervention group, 156 medication changes for drug overuse and drug underuse persisted 6 months after admission compared to 59 in the control group (adjusted rate ratio 1.22 [95%CI 1.01-1.49] *p* = 0.039).

**Conclusion:**

Emergency department pharmacists do contribute to reduction of drug overuse and drug underuse of medication in patients with a hospitalisation related to adverse drug events after an emergency department visit.

## Background

Medication has many benefits, but may also lead to adverse drug events (ADEs). These are defined as potentially harmful events related to the use or misuse of medication. Worldwide ADEs contribute to 5-7% of Emergency Department (ED) visits resulting in hospital admissions [1–4]. In approximately 25% of patients with such a ADE-related hospitalisation drug overuse and in approximately 50% drug underuse was the cause [5]. Drug overuse (prescribed medication without an indication) and drug underuse (omission of prescribed medication despite an indication) can compromise patient safety and may lead to ADE-related readmissions [6]. Clinical pharmacists are specialized in recognition of ADEs and tackling drug overuse and drug underuse [7]. Implementation of a clinical pharmacist in the ED may therefore improve patient safety. As 38 to 65% of ADEs are not recognized in EDs by physicians, ED pharmacists could also have a role in identifying these high risk patients [2, 5, 8–10]. Studies already found that ED pharmacists frequently detect drug overuse and drug underuse during medication reviews [9, 10]. However, these studies did not investigate the persistence of medication changes after hospitalisation. Therefore, the primary aim of this study was to determine the effect of an ED pharmacist on the number of medication changes for drug overuse and drug underuse that persisted 6 months after discharge in patients with an ADE-related hospitalisation after an ED visit. The secondary aim was to determine the effect on: the number of all medication changes during admission and 6 months after discharge, recognition by physicians in the ED of ADEs causing hospitalisations, patient satisfaction with the intervention, and the number of ADE-related readmissions.

## Methods

### Study design

This prospective multicenter non-randomized controlled intervention study was conducted between October 2016 and January 2018 in the 1320-bed Erasmus University Medical Center in Rotterdam and in the 550-bed general teaching OLVG hospital in Amsterdam in The Netherlands. The study protocol received a waiver from the Medical Ethics Committee as it was outside the scope of the Human Research Act (MEC-2016-346). All data were handled according to the European General Data Protection Regulation. Written informed consent was obtained from all included patients at admission.

### Selection of participants

Patients aged 18 years and older were eligible for inclusion if they were hospitalised for more than 24 hours after visiting the ED. Patients were excluded based on the following criteria: no communication possible due to critically ill condition or language barrier; cognitive impairment; transfers to other hospital; no pre-admission medication; admission due to problems with cancer treatment; alcohol intoxication and/or (self) poisoning related hospitalisation; psychiatric hospitalisation; intensive care unit (ICU) hospitalisation; foreign tourist or homeless patient; already included patient readmitted within the research period; and patients with no informed consent.

Patients with a potential ADE-related cause of admission were included in the intervention group, and patients with an unlikely ADE-related cause were included in the control group. The prevalence of ADE-related hospitalisations after an ED visit is 5-7% worldwide, leaving 93-95% eligible for inclusion as control patients. Control patients were included on the same day and from the same ward as the intervention patient. They were selected in order of appearance on the screening list. The ideal control group should consist of patients with an ADE-related hospital admission and where the ED pharmacist did not interfere. This was not possible as we know that a large part of ADE-related hospitalisations are not recognised as such in the ED [2, 5, 8–10]. It is considered unethical to deliberately withhold such important information for research purposes as this may compromise patient safety.

### Study procedures

Two equally trained ED pharmacists screened all patients who were hospitalised after an ED visit and who met the inclusion criteria for ADE-related hospitalisations. The additional training of the ED pharmacists included certified courses in recognition of ADEs and in performing medication reviews, and training on the job by senior clinical pharmacists. The Hospital Admissions Related to Medication (HARM)-trigger list as well as registered drug information was used to select potential ADE-related hospitalisations [11]. Patients were included in the intervention group when an association of the reason of admission with medication was determined as “possible” (score 0 to 4) using an adjusted version of Kramer’s causality algorithm and agreed upon by three assessors: the ED pharmacist, a senior clinical pharmacist, and the treating physician [4, 12]. Patients were assigned to the control group in case of an unlikely causal association (score − 4 to − 1). Patients in the control group received their usual pharmaceutical care. This consisted of medication reconciliation at admission by pharmacy technicians who verified pre-admission medication with patients using information from community pharmacies. Within the OLVG hospital medication reconciliation was also performed at discharge. Clinical pharmacists oversee pharmacy technicians during their activities. Also, computerised medication surveillance alerts (e.g. interactions, duplicate medication, underdosing, overdosing) were monitored and processed on a daily basis by clinical pharmacists in both hospitals during admission.

The intervention of the ED pharmacists consisted of identification of ADE-related hospitalisations, a pharmacist-led medication review, patient counselling and communication to the patient’s general practitioner and community pharmacy after discharge. The pharmacist-led medication review was performed according to the Systematic Tool to Reduce Inappropriate Prescribing to optimise therapeutic effect and minimise (potential) drug related problems (DRPs) [13]. This included a medication interview with patients and an analysis which resulted in recommendations for medication changes to solve DRPs. These recommendations were discussed with the patients hospital physician which could result in additional medication changes during admission or recommendations for primary care. Patient counselling focused on the causative agent of the admission and any medication changes due to drug overuse and drug underuse. The “teach-back” method was used in which the patient was encouraged to repeat the message of the caregiver, to aid patient empowerment [14]. Information regarding the reason for admission, the pharmacist-led medication review, medication changes, and recommendations that needed follow-up, were transmitted in a discharge letter to the patient’s general practitioner and community pharmacy to ensure continuity of care.

### Data collection

Patient and medication data were collected from the hospital electronic patient record system (HiX; Chipsoft, Amsterdam, The Netherlands and Epic System Corporation; Verona, Wisconsin, USA). The Charlson Comorbidity Score was assessed [15]. The medication overviews obtained after medication reconciliation at admission were used to assess the number of chronic medicines. Patients received a questionnaire at inclusion to assess health literacy, medication adherence, and their living situation (classified as independent, dependent at home or dependent in a nursing home). The Health Literacy Score (HLS) was determined with the health literacy questionnaire, a score of > 2 indicates an adequate health literacy [16]. Medication adherence was determined with the Medication Adherence Rating Scale (MARS) questionnaire, a score of 0-19 indicates medication non-adherence [17].

A DRP was defined as an event or circumstance involving drug therapy that actually or potentially interferes with the desired health outcome. DRPs were categorised as (1) drug overuse, (2) drug underuse, (3) contraindication, or (4) other (Table 1) [18]. Each DRP was categorised by both ED-pharmacists separately. When they disagreed, the categorisation was discussed within the research group until a consensus was reached. Medication changes were defined as changes in medication during admission because of a DRP. In the control group, these medication changes were initiated by physicians. In the intervention group, they were initiated by physicians either by themselves or based on recommendations of the ED pharmacist.

**Table 1:** Definition per category of drug related problems

Definition	Example
**Drug overuse**	
Drug without indication	Using a protonpumpinhibitor with no indication
Drug dose too high	RAAS-inhibitor dose too high for hypertension in older patient with low blood pressure
Inappropriate duplication of therapeutic group or active ingredient	Double platelet aggregration inhibitors without indication for double treatment
Too many drugs prescribed for indication	Four antihypertensive agents while blood pressure is low
**Drug underuse**	
No drug treatment in spite of existing indication	No laxative agent in combination with chronic opioid treatment
Drug dose too low	Simvastatin dose is 10 mg once daily while patient has no indication for a low (insufficient) dose
Duration of treatment too short	Antibiotic prophylaxis treatment is stopped while patient still has high risk of infections
**Contraindicated**	
Inappropriate drug according to guidelines/formulary	Glimepiride for diabetes mellitus type 2 instead of gliclazide (first choice of sulphonylureaderivative according to national guideline)
Inappropriate drug (within guidelines but otherwise contra-indicated)	Simvastatin instead of rosuvastatin for patient with non-adherence with evening dose
Inappropriate combination of drugs	Tramadol in combination with fluoxetine
**Other**	
Adverse drug reaction	Pulmonary embolism because of olanzapine
Inappropriate drug form (for this patient)	Tablet too big for patient with swallowing problems
No or inappropriate outcome monitoring (including therapeutic drug monitoring)	Potassium level too low in patient using high dose of thiazide and no monitoring on electrolytes in the past year.

The number of medication changes processed during admission, was obtained from the hospital’s electronic patients records. Medication overviews from community pharmacies were obtained 6 months after index admission to determine the persistence of medication changes. The recognition of ADEs as reason of hospitalisation in the ED was analysed by using ED visit reports from ED physicians. Recognition of ADEs was positive if the causative medication was mentioned as such in the ED visit report from the ED physician.

The degree of patient satisfaction with the intervention of the ED pharmacist was determined in the intervention group by mailing a Visual Analogue Scale (VAS) 3 months after admission. The VAS had a 100 mm line on which the patient marks the degree of satisfaction with the intervention of the ED pharmacist. The VAS value was the measured distance from the origin of the line. Data on hospital admissions of intervention patients 6 months before and 6 months after the index admission were obtained from general practitioners. Both ED-pharmacists assessed these admissions for ADE-related causes and potential preventability separately, and met to reach consensus when they disagreed. To determine the potential preventability of these ADE-related admissions an adjusted algorithm of Schumock was used [4, 19]. All data were processed with Open Clinica (Open Clinica LLC version 2.1, Waltham, USA).

### Outcome measures

Primary outcome was the number of medication changes for drug overuse and drug underuse that took place during hospital admission and persisted 6 months after hospital discharge. Secondary outcomes were the number of all medication changes that took place during admission and persisted 6 months after discharge, the number of medication changes for drug overuse and drug underuse that took place during admission and all medication changes during admission, the proportion of ADEs causing hospitalisations recognised by physicians in the ED, the degree of patient satisfaction with the intervention of the ED pharmacist, and the number of (ADE-related) hospitalisations within a period of 6 months before and after the index admission within the intervention group**.**

### Data analysis

In each group 100 patients were planned to be included. Assuming 60% of these would be eligible to evaluate medication changes at 6 months after discharge with a coefficient of variation of 1.5, the power to detect a ratio of means of 1.5 would be 52%, increasing to 84% for a ratio of 1.8. Analyses were performed with IBM SPSS version 25.0 (IBM software, New York, USA) and with SAS 9.4 (SAS Institute Inc., Cary, NC, USA). Continuous variables regarding patient characteristics were tested for normality with the Shapiro-Wilk test. Differences in patient characteristics between both groups were determined with appropriate tests (t-test, Mann-Whitney U test or Pearson’s Chi-Square test). A Poisson regression analysis with robust standard errors was used to estimate the difference in number of medication changes for drug overuse and drug underuse that persisted 6 months after discharge between the intervention group and the control group, for all medication changes, and for the number of medication changes during admission. The main analysis was performed using the last observation carried forward for missing data for patients who died before the end of the follow up period and was adjusted for number of chronic drugs. A sensitivity analysis was performed with exclusion of these patients and with adjustment for patient characteristics which differed significantly between both groups. The number of medication changes during admission was used as offset. Adjusted rate ratios for the number of chronic drugs, 95% confidence intervals (95% CI), and *p*-values were reported (*p* < 0.05 was considered to be statistically significant). Descriptive statistics were performed to assess the proportion of ED visits recognised by physicians as being caused by medication, and to describe patient satisfaction with the intervention. A within patient pre-post design was used for the outcome of readmissions in the intervention group. Difference in number of (ADE-related) hospitalisations between the period of 6 months before the index admission and 6 months after the index admission in intervention patients was determined by the Wilcoxon signed rank test.

## Results

### Study population

**Fig. 1:**
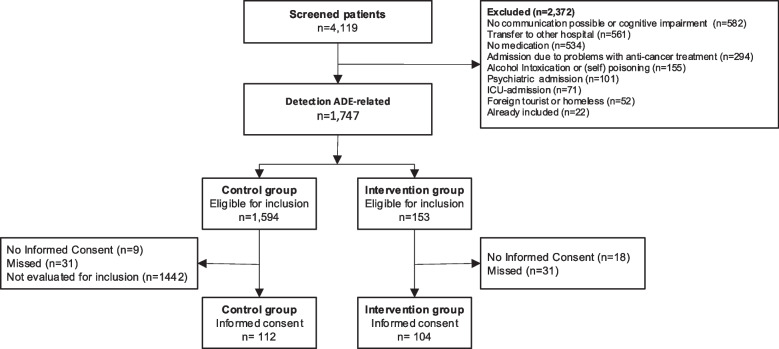
Study flowchart

A total of 4119 patients were screened for eligibility during the study period (Fig. 1). The most common reason for patient exclusion in the control group was due to selection in a 1:1 ratio. In the intervention group this was due to impossible communication because of the condition of the patient or because of a language barrier. In total, 216 patients were included of which 112 were in the control group and 104 were in the intervention group (Fig. 1). Intervention patients were comparable to control patients, except for health literacy which was significantly lower in the intervention group (Table 2).

**Table 2:** Characteristics of included patients

Characteristic	Control (***n*** = 112)	Intervention (***n*** = 104)	***p***-value
**Age (years),** Median [IQR]	67 [54-77]	68 [57-78]	0.63^*^
**Sex female,** n (%)	57 (50.9)	54 (51.4)	0.88^†^
**Hospital type (University Medical Center**), n (%)	54 (48.2)	47 (44.7)	0.61^†^
**Length of hospitalisation (days)**, median [IQR]	5 [3-9]	6 [3-9]	0.95^*^
**Main specialty during hospitalisation,** n (%)			0.14^†^
Surgical	30 (26.8)	19 (18.3)	
Internal Medicine	82 (73.2)	85 (81.7)	
**Charlson Comorbidity Score,** median [IQR]	1.0 [0.0-2.0]	1.0 [0.0-2.0]	0.53^†^
**Renal function**, mean ± SD	71.5 ± 28.6	65.9 ± 27.8	0.16^‡^
**Number of chronic drugs at admission,** mean ± SD	7.1 ± 4.3	8.0 ± 4.7	0.14^‡^
**Medication non-adherence** (MARS < 20**)**, n (%)	15 (13.4)	9 (8.8)	0.29^†^
**Living situation,** n (%)			0.23^†^
Independent, home	98 (87.5)	83 (79.8)	
Dependent, home	9 (8.0)	16 (15.4)	
Dependent, nursing home	5 (4.5)	5 (4.8)	
**Health Literacy Score**, median [IQR]	3.3[2.7-4.0]	3.0 [2.3-4.0]	0.03^*^

*IQR* interquartile range, *SD* standard deviation

^*^Based on Mann-Whitney U test

^†^Based on Pearson Chi-square test

^‡^Based on Independent T-test

### Medication changes

**Fig. 2:**
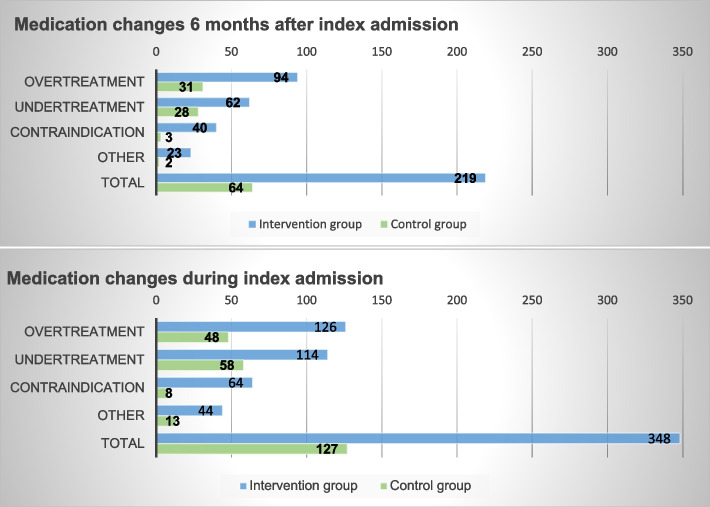
Number of medication changes for drug related problems per category at moment of admission and six months after hospital discharge (persisted medication changes)

Table 3 shows the number of medication changes related to drug overuse and drug underuse that persisted 6 months after discharge. In the control group, 59 of the initial 106 medication changes for drug overuse or drug underuse were persisted 6 months after discharge and in the intervention group 156 of the initial 240, resulting in an adjusted rate ratio of 1.22 [95% CI 1.01-1.49]. Also, the number of medication changes for all DRP categories during admission were more frequent and persisted more often in the intervention group at 6 months after discharge (see Table 3). In the intervention group, nine patients died after a mean follow-up of 3.0 months and in the control group six patients died (mean follow-up 2.7 months). Excluding these patients in sensitivity analysis did not affect the results substantially compared to the main analysis. The rate ratio also did not change when we adjusted for Health Literacy Score. Figure 2 shows the number of medication changes during hospital admission and the number of medication changes persisted 6 months after hospital discharge in each group per DRP category (i.e. drug overuse, drug underuse, contraindication, other).

**Table 3:** Number of medication changes for drug related problems (DRPs) six months after hospital discharge and at moment of admission

	Control group (***n*** = 112)	Intervention group (***n*** = 104)	Rate ratio [95%CI]	***p***-value
**Six months after hospital discharge**				
Related to DRPs drug overuse and drug underuse, n (% of DRPs during admission)	59 (55.7)	156 (65.0)	1.22 [1.01-1.49]	0.039
Related to all DRPs, n (% of DRPs during admission)	64 (50.4)	219 (62.9)	1.29 [1.07-1.56]	0.009
**During admission**				
Related to DRPs drug overuse and drug underuse, n	106	240	2.31 [1.73-3.10]	< 0.0001^*^
Related to all DRPs, n	127	348	2.83 [2.20-3.63]	< 0.0001^*^

^*^adjusted for number of chronic drugs

### ADE-recognition, patient satisfaction, readmissions

ED physicians recognised the causal ADE in 59.1% as reason for admission (see Table 4). The response to the patient satisfaction questionnaire was 58%. The median patient satisfaction score was 80 [IQR 75-90]. There was no significant difference between the number of ADE-related hospitalisations in 6 months before compared to the 6 months after index admission in the intervention group (*p* = 0.554). This was also the case for the total number of hospital readmissions and the number of preventable ADE-related hospitalisations (*p* = 0.995, *p* = 0.291 respectively) (Table 5).

**Table 4:** Characteristics of the most frequently occurring unrecognized ADE-related hospitalisations

	Reason of admission	Unrecognized/total n (%)	Involved drug group (n)	DRP (n)
**1**	**Fall**	18/21 (86)	Benzodiazepines (7)	drug overuse (4), contraindication (2), ADR^a^ (1)
			Antihypertensives (7)	drug overuse (5), contraindication (2)
			Antidepressants (2)	drug overuse (2)
			Opioids (2)	drug overuse (2)
**2**	**Electrolyte and Fluid disorders**	5/9 (56)	Diuretics (4)	ADR^a^ (3), contraindication (1)
			Protonpumpinhibitors (1)	ADR^a^ (1)
**3**	**Cerebro Vasculair Accident (CVA)**	4/8 (50)	Statins (2)	drug underuse (2)
			Trombocyte aggregation inhibitors (2)	drug underuse (1), other (non-adherence) (1)

^a^Adverse Drug Reaction

**Table 5:** (ADE-related) hospitalisations in intervention group 6 months before admission and 6 months after hospital discharge

	T = 6 months before admission (***n*** = 95)	T = 6 months after discharge (***n*** = 95)	***p***-value^*^
**Hospital admissions,** n	57	54	0.995
ADE-related hospital admissions, n (% of hospital admissions)	21 (37)	17 (32)	0.554
Preventable ADE-related hospital admissions, n (% of ADE-related hospital admissions)	14 (67)	8 (47)	0.291

^*^Based on Wilcoxon signed rank test

### Post-hoc analysis

Since our control group was different from the intervention group with respect to ADE-relatedness of the admission, and the main analyses yielded a significant difference, we performed the Poisson analysis in subgroups as post-hoc analysis (see Table 6). In the first post-hoc analysis we analysed the difference in medication changes for drug overuse and drug underuse that persisted 6 months after discharge, between the subgroups “non-recognised ADE-related hospitalisations” and “recognised ADE-related hospitalisation” both within the intervention group. This resulted in no significant difference for any outcome. In the second post-hoc analysis we performed the same analysis for the subgroups “non-recognised ADE-related hospitalisations” (part of intervention group) and the control group, resulting in a significant difference between these two groups (Methods section).

**Table 6:** Post-hoc analysis of number of medication changes for DRP categories drug overuse and drug underuse six months after hospital discharge (primary outcome) for subgroups

	**Intervention group non-recognised (*n*** **= 36)**	**Intervention group recognized (*n*** **= 63)**	**Rate ratio [95%CI]**	***p*-value**
**Six months after hospital discharge**				
Medication changes related to drug overuse and drug underuse, n	57	83	0.94 [0.80-1.10]	0.424^*^
	**Intervention group non-recognised (*n*** **= 36)**	**Control group (*n*** **= 104)**	**Rate ratio [95%CI]**	***p*-value**
**Six months after hospital discharge**				
Medication changes related to drug overuse and drug underuse, n	66	59	1.30 [1.05-1.61]	0.014^*^

^*^adjusted for number of chronic drugs

## Discussion

Our study showed that by implementing ED pharmacists, significantly more medication changes for drug overuse or drug underuse persisted 6 months after hospital discharge in patients with an ADE-related hospitalisation after an ED visit compared to usual care. Only 59.1% of the ADE-related hospitalisations were recognised as such by ED physicians, and patient satisfaction with the ED pharmacist was high. No effect was seen on ADE-related readmissions within 6 months after the intervention.

Our findings confirm the results of previous studies which looked into the effect of clinical pharmacist-led medication reviews in acutely admitted patients where a range of 53-267 medication changes for DRPs per 100 patients were reported. In our study we found 348 medication changes for DRPs in the intervention group (104 patients) [20–22]. Also, these studies found that most of the medication changes for DRPs were due to drug overuse and drug underuse (51-65%) which is in line with our findings (69%). Previous studies did not investigate the persistence of the medication changes for DRPs and did not use control groups [20–22]. Although we did use a control group, it is different from the intervention group with respect to medication relatedness of the admission. This may cause some bias, as physicians may be more likely to intervene on the medication in case of ADE-related hospitalisations. If the possibility of bias is true, we would expect a higher number of medication changes in the subgroup of patients in which the doctor recognised the admission as ADE-related, compared to the non-recognised group (both within the intervention arm). In contrast, the post-hoc analysis did not show a significant difference between these subgroups. In addition, the number of medication changes in the non-recognised group within the intervention arm was not comparable to the number in the control arm of the study implying that the increased number of medication changes in the non-recognized intervention group was not because of the awareness of the physicians of the ADE, but more likely because of the intervention. We also showed that in only 59% of the intervention patients, the reason for admission was recognised as ADE-related in the ED. This corresponds with previous studies where 35-62% of ADE-related hospitalisations were recognized as such by ED physicians [2, 8, 23, 24].

The median patient satisfaction score of 80 [IQR 70-90] with the intervention of the ED pharmacist was comparable with previous similar reported scores for interventions of clinical pharmacists [25]. This high score might be explained by providing useful information about their medication and the face-to-face time the pharmacist spent with the patient. This high satisfaction score shows the impact of pharmacist involvement on a patient-related outcome implying societal relevance.

To our knowledge this was the first prospective controlled intervention study that showed the effect of ED pharmacists in patients with an ADE-related admission, which especially looked into the persistence of medication changes for DRPs and also reported patient satisfaction. In addition, the study was conducted in both a general teaching hospital and a university hospital contributing to generalisability of the results. In contrast to other studies, the intervention of the ED pharmacists was an interdisciplinary intervention including patient counselling and medication information transfer to the next healthcare providers. Also, we performed a sensitivity analysis to test the influence of patients lost in the follow-up period. A follow-up consultation with the patient and the ED pharmacist or the patient’s general practitioner is suggested for continuity of care and to further improve the sustainability of medication changes.

Our study has three important limitations. First, control patients were hospitalised after an ED visit without an ADE-related reason for admission. This could have limited the number of medication changes for DRPs in the control group. The post-hoc analyses showed that this probably did not introduce bias. Secondly, the non-randomized design may have introduced bias. And third, the exclusion of patients where no communication was possible due to critically ill condition or language barrier may have introduced selection bias, as it can lead to exclusion of the most serious ADEs.

## Conclusion

This study showed that implementation of ED pharmacists resulted in a significant reduction of drug overuse and drug underuse of medication in patients with an ADE-related hospitalisation after ED visit and that these medication changes persisted 6 months after discharge. Additionally, DRPs of all categories were reduced and ED pharmacists contribute to recognition of ADEs as reason of admission in the ED. Patients were satisfied with the intervention.

## Data Availability

The datasets generated and analyzed during the current study are not publicly available because they contain information that could compromise the privacy of research participants, but are available from the corresponding author upon reasonable request.

## Abbreviations

ADEs: Adverse drug events
CCS: Charlson comorbidity score
DRP: Drug related problem
ED: Emergency department
HARM: Hospital admissions related to medication
HLS: Health literacy score
ICU: Intensive care unit
IQR: Interquartile range
MARS: Medication adherence scale
